# A comparative framework to analyze convergence on Twitter electoral conversations

**DOI:** 10.1038/s41598-022-21861-6

**Published:** 2022-11-09

**Authors:** Daniel Cárdenas-Sánchez, Andrés Miguel Sampayo, Maykol Rodríguez-Prieto, Alejandro Feged-Rivadeneira

**Affiliations:** 1 Escuela de Ingeniería, Ciencia y Tecnología de la Universidad del Rosario, Bogotá, Colombia; 2 Facultad de Estudios Internacionales, Políticos y Urbanos de la Universidad del Rosario, Bogotá, Colombia

**Keywords:** Applied mathematics, Statistics, Psychology and behaviour

## Abstract

Literature on social networks and elections has focused on predicting electoral outcomes rather than on understanding how the discussions between users evolve over time. As a result, most studies focus on a single election and few comparative studies exist. In this article, a framework to analyze Twitter conversations about the election candidates is proposed. Using DeGroot’s consensus model (an assumption that all users are attempting to persuade others to talk about a candidate), this framework is useful to identify the structure and strength of connections of the mention networks on the months before an election day. It also helps to make comparisons between elections and identify patterns in different contexts. In concrete, it was found that elections in which the incumbent was running have slower convergence (more closed communities with fewer links between them) and that there is no difference between parliamentary and presidential elections. Therefore, there is evidence that the political system and the role of the incumbent in the election influences the way conversations on Twitter occur.

## Introduction

Understanding the heterogeneity of political opinion and the formation of cultural consensus in the context of presidential elections is one of the quintessential questions in studies of democratic processes. Social networks, like Twitter and Facebook, have disrupted mass media’s role in political processes to the degree that both activists and academics advocate for the regulation of these technologies and even the term Weapons of Math Destruction (WMD) has been coined to describe their potential threat to democracy^1–3^. Usage and expenditure of political campaigns in social networks has outpaced regulatory frameworks and knowledge about their role and potential^1–4^. While a significant amount of the growing body of literature that explores the role of social media on elections has focused on the possibility of predicting outcomes with a plethora of methods^5,6^, the possibility of developing robust methods for this purpose has been disputed and controversial^7^. Simultaneously, social network analysis remains underused as a paradigm to understand relational phenomena in political science^8,9^. In this study, the use of a simple method is explored, based on social network analysis (convergence), to understand the formation of consensus in political debates (elections).

### Twitter studies

As mentioned above, studies using data from social networks such as Twitter to understand electoral processes have focused mostly on the possibility to predict outcomes without consensus^5–7^. One of the biggest limitations in this line of inquiry is the focus on single election points without comparing different elections within a country or across countries to study the effect of context. Furthermore, there is no unified methodology to capture (search terms), clean, process and analyze data (mention networks vs. retweet networks, for example), which results in a body of literature with conflicting findings and a lack of generalizable conclusions. Many of these papers are authored by engineers, mathematicians and similar professionals in quantitative disciplines who often lack awareness of the questions, debates and phenomena studied by political scientists, resulting often in isolated papers that propose new methods to describe one aspect of a single election. There is a clear need for comparable and descriptive studies across time and context to better understand the multiplicity of findings. The overarching narrative, however, is that Twitter is crucial and unique both for political strategy and electoral studies. Additionally, it is important to note that Twitter is among the few social networks (if not the only, with millions of users all around the world) that allows its relational data, like the structure of interactions, for the academic use and analysis.

A different line of work with Twitter data, relevant to this study, has addressed political polarization on the social network, but not necessarily electoral outcomes, rather focusing broadly on phenomena such as manipulation, the effects of coordinated behavior and disinformation campaigns^10–12^. These studies find that coordinated behavior both for disaster response and disinformation campaigns affects the structural properties of the social network and therefore influences the flow of information^10–13^. Importantly, these studies (among others) show that disinformation campaigns exacerbate political polarization, resulting in problems of coordination and even in physical violence between groups with opposing political views^10–13^. Some of these studies have been produced by research groups of multidisciplinary nature, which combine qualitative and quantitative methods to characterize interactions on the social network and compare data from different countries, political processes as well as natural disasters^10–13^.

### Twitter and elections

Twitter has become instrumental for the political functioning of democracies, especially during elections. It is used by campaigns broadcasting their candidate’s messages and interacting directly with possible voters, which according to studies in the UK and the Netherlands has a positive effect on winning more votes^14,15^. Consequently, there has been some academic exploration into how elections can be influenced by social media interactions between citizens, specifically on the way they relate to election results. This relationship has been explored in descriptive studies on specific contexts, like an analysis on the performance of US presidential candidates in the 2016 election^16^. Nonetheless, investigations have not gone beyond descriptions of a single electoral context.

### Consensus and second eigenvalue

The idea of using spectral theory to analyze social phenomena such as consensus has been widely used. For example, Cultural Consensus Theory (CCT) and Analysis (CCA) have been implemented in studies to explore beliefs about disease^17^, political knowledge^18^, and more recently, the role of the State in information and polarization in mass media outlets^19^ These studies all rely on representing different responses to specific questions as matrices, and calculating the ratio of the first to second Eigenvalues. The purpose of CCA is to infer individuals who are more accurately informed about the social norms without externally defining something as “truth”^20^. Another example of spectral analysis on the formation of opinions is the use of formal models and validation through empirical analysis of Strategic Networks (a combination of Social Network Analysis and Game Theory)^21,22^. These studies assume that individuals are trying to persuade other agents of a network, and that agents update their preferences or opinions over time. They both use spectral analysis, mainly the ratio of the first and second Eigenvalues, to understand the formation of opinion and how network structure affects it.

Overall, spectral graph theory seeks to identify and measure properties of graphs that are related to the eigenvalues and eigenvectors of its matrix representations. One of the reasons why this approach can be useful is that while describing a graph with all its details entails listing all present links among its approximately $n^2$ dyads, it can have at most n different eigenvectors and associated eigenvalues.

Such measures have been applied to the analysis of a wide variety of social phenomena that can be aptly described using graphs and networks. For instance^23^, rely on the smallest eigenvalue of the adjacency matrix in order to characterize behaviors in games of strategic substitutes, such as public good games. This eigenvalue turns out to be useful because it captures the extent to which the graph is bi-patrtite or close to bi-partite, which is the structural feature of the network determining whether best-response dynamics of different players tend to amplify each other, leading to multiplicity and instability of equilibria, or whether instead they tend to attenuate each other, leading to uniqueness and stability. In a very different vein^24^ show that the second largest eigenvalue of normalized adjacency matrices index the speed of convergence in the widely applied DeGroot model of diffusion. This particular eigenvalue turns out to be important in these models because it reflects the extent to which the network is close to involving multiple components instead of being strongly connected. The beauty and power of spectral graph theory therefore hinges on its ability to identify network statistics which reflect key structural features which can explain similar behavioral outcomes on otherwise very dissimilar networks. For a broad survey of spectral graph theory see^25^.

### A framework to analyze consensus formation on Twitter

This paper proposes a methodology to assess the evolution and the structure of Twitter interactions, specifically, it aims at discovering patterns based on the mentions of candidates in political elections. To achieve this, Twitter is seen as a network where each user is competing for attention. This means that each tweet seeks other users’ reactions expressing their opinions around a single topic and, in the case of elections, around a specific aspect of a candidate. Therefore, the interactions are not of a approve/disapprove nature (like the studies attempting to predict the outcome of elections suggested), but rather built on the idea that the public should talk more about one or more of the candidates.

Each user of the network can persuade others in one of two ways. The first one is directly: by writing a tweet mentioning a candidate, or answering a tweet with the same characteristics (this includes tweets written by a candidate). Writing is a way to propose a new topic of conversation, while answering is veering an existing conversation in a new direction. The second way is indirectly: by interacting with a tweet, clicking fav or retweeting it, which is an expression of support for the current conversation and an attempt to make it more prominent for the rest of the public. As a result, the success of each direct action can be measured by how many indirect interactions the tweet has, since it is an indicator of the public’s attention. This framework, even when it simplifies the interactions on Twitter, is useful because it allows researchers to explore the changes in the general conversation, the creation of communities discussing each of the candidates, and the combination of both, the convergence of the network (or its ability to reach a consensus around one topic of discussion). There are many alternatives that are not considered here to make the analysis simpler and comparable between countries. For instance, a user could answer a tweet with a message of support to the original and not necessarily with a new or slightly different issue to discuss (an indirect interaction done directly). In the same way, someone could retweet a message to change the focus it had (a direct action done indirectly). However, this paper assumes that these situations are exceptional and that the general behavior is as expected. Otherwise, it will make comparisons between different countries and languages.

In practice, this analysis requires the creation of graphs in which the users that participated in the conversation are nodes, the mentions between them are edges, and the latter’s weight is a sum of the tweet’s favs and RTs. To compare elections of different countries, a single criterion was used to form the graphs: using tweets where at least one of the three main candidates was mentioned. This means that, by formation, the graphs have three dominant nodes because all the other nodes will be attached to at least one of them, resulting in a formation like the Barabasi graph theory. On each network, analyses on the modularity (structure), efficiency (connections between nodes) and second eigenvalue (convergence) are performed. These three measures give a sense of how polarized the conversation is and how likely diverse messages can propagate through the network.

Using the basis of DeGroot^21^ theory, the second eigenvalue is taken as evidence of convergence even if the conditions on this study are not the same as DeGroot’s theoretical models. To supply this, a series of simulations are done to prove that convergence—seen in practical terms as a measure of how fast can information flow through a network of communities—depends on the structure and connections of the graph. In other words, the second eigenvalue is related to the modularity and efficiency of the network.

In the election framework, two analyses are performed, one of each election and its evolution before the election day, and another between elections. Therefore, each election is divided monthly, starting from the fifth month before election day, and a graph is created for each of them. In this way, the evolution of the consensus measure can be appreciated. Furthermore, to compare between elections two hypotheses are tested:

H1: Elections where the incumbent is running have a faster consensus

The incumbent has a predominant role in defining the agenda for public debate. Therefore, elections with the incumbent running are seen as a “referendum” on whoever is in power28. As a result, the central issue to discuss is the performance of the incumbent, which will lead to a centralized network around the candidate and, therefore, a higher consensus.

H2: Parliamentary elections have a faster convergence than presidential

In parliamentary elections, each party’s candidate for Prime Minister could be the principal factor in the campaign, but it is not the only one. Since the party is as relevant as the candidate, more issues than his or her merits are discussed and, therefore, more connections with other political affiliations are expected.

To test them, data from elections held in 2020, 2021 or the latest from countries with the biggest reported Twitter usage in 2021 was collected. In these 35 elections, a president or main parliament was elected and all three of the most voted candidates had an official Twitter account. In parliamentary elections, the party leader’s account was used.

## Results

Theoretical properties described in graph theory are useful to analyze how the efficiency of learning and diffusion depend, in sensitive ways, on the way the social network is organized^26^. On each network (each election and month before election day), analyses on the modularity (structure), efficiency (connections between nodes) and second eigenvalue (convergence) were performed. These three measures give a sense of how polarized the conversation is and how likely diverse messages can propagate through the network. Modularity is used as a quality index for clustering, which means that networks with higher modularity (closer to one) have dense connections between the nodes within modules but sparse connections between nodes in different modules^27^. On the other hand, efficiency is a measure that refers to how a network exchanges information between nodes. It is inversely proportional to the shortest path; a greater distance between two nodes means less efficiency. For instance, if the distance between two nodes is 1 (meaning that they are connected directly) the efficiency is 1, while if the distance between two nodes is 2, the efficiency will be 0.5^28^. Finally, as mentioned before, the second largest eigenvalue has a relationship with the magnitude of the speed of convergence on a network in which every node had to make a one-dimensional decision^29^ like an election.

**Figure 1:**
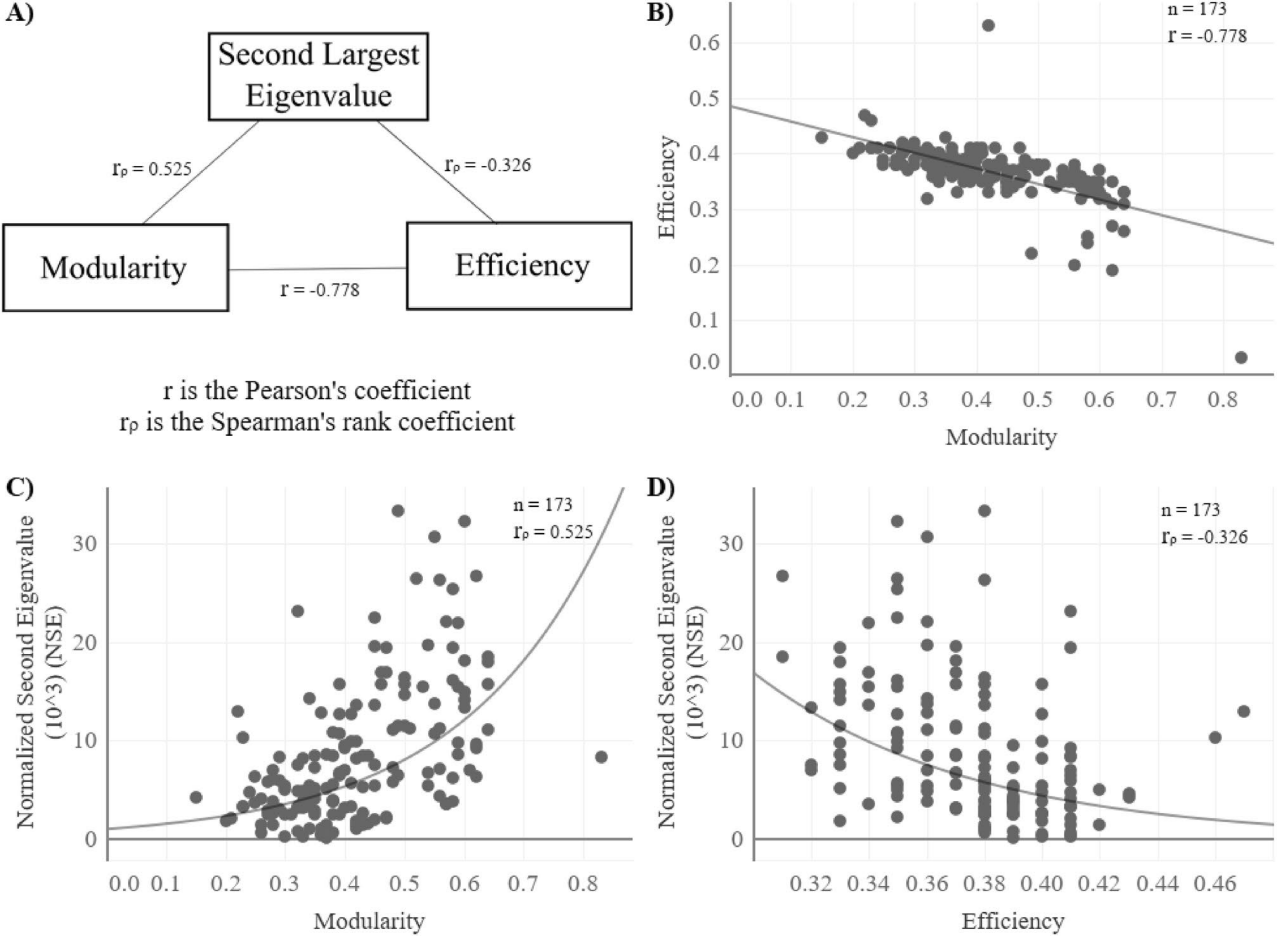
Correlations coefficients of all elections and months (there are five points per election) between: (**A**) the three measures (Spearman’s rho reported for non-linear relationships), (**B**) modularity and efficiency (extreme values removed in the graph for better understanding), (**C**) modularity and the second eigenvalue (extreme values removed in the graph for better understanding), and (**D**) efficiency and the second eigenvalue (extreme values removed in the graph for better understanding).

**Figure 2:**
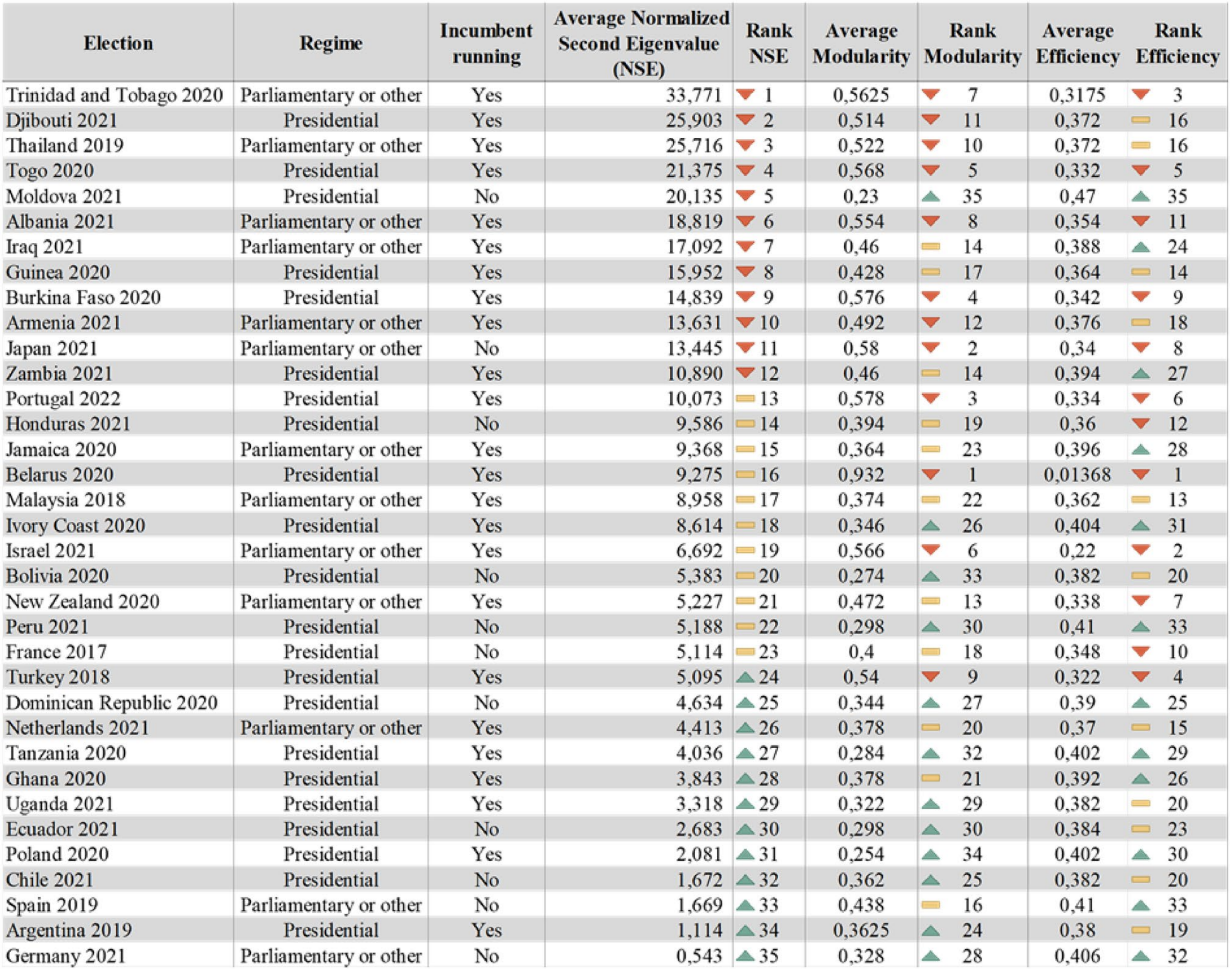
Average of the measures by election. The icons on the rankings are placed according to the effect of the variable on the convergence (higher values of the second eigenvalue are related with a slow convergence, like high values of modularity and lower values of efficiency).

Therefore, the Twitter data for each election (downloaded through the Twitter API) was divided into five periods. Then each period, which represents a month before election day, was converted into a graph (using the igraph package in R) to find the mentioned measures. To obtain the second eigenvalue the graph was first converted into an adjacency matrix after its eigenvalues spectrum was found and then, sorting by its magnitude in absolute, the second highest is obtained. The modularity was obtained with an igraph function called “modularity(g)” and the efficiency was calculated by building a function based on its theoretical definition. As mentioned before, modularity and efficiency are correlated to the second eigenvalue’s behavior and each other (Fig. 1A). The latter is a negative relation: when modularity increases, efficiency tends to decrease (Fig. 1B). Also, higher levels of clustering on the network are related with higher values of the second eigenvalue (slower convergence) (Fig. 1C), and the higher the efficiency in a graph, the faster it will reach a consensus (in other words, the smaller the second eigenvalue) (Fig. 1D). The average results for each election are presented in Fig. 2.

At this point, it is worth mentioning that efficiency and modularity are not the only measures related to the second eigenvalue, nor is there a causal relationship between them. Figure 1 shows that the three measures are related to each other in the proposed direction and, therefore, can be used to describe the polarization of an electoral conversation (understood as echo chambers where only one version of the arguments is listened to^30^). In this sense, modularity measures how divided the network is in groups, efficiency shows how easy it is to spread a message through all the nodes, and the second eigenvalue shows how likely, if all the connections remain equal, it will be for the network to reach a unanimous consensus around a candidate. Other measures, like the betweenness (a measure of the importance of certain nodes on passing information^31^), can be related to the second eigenvalue, however, they are beyond the scope of this study since it is focused on the general structure of the conversation.

**Figure 3:**
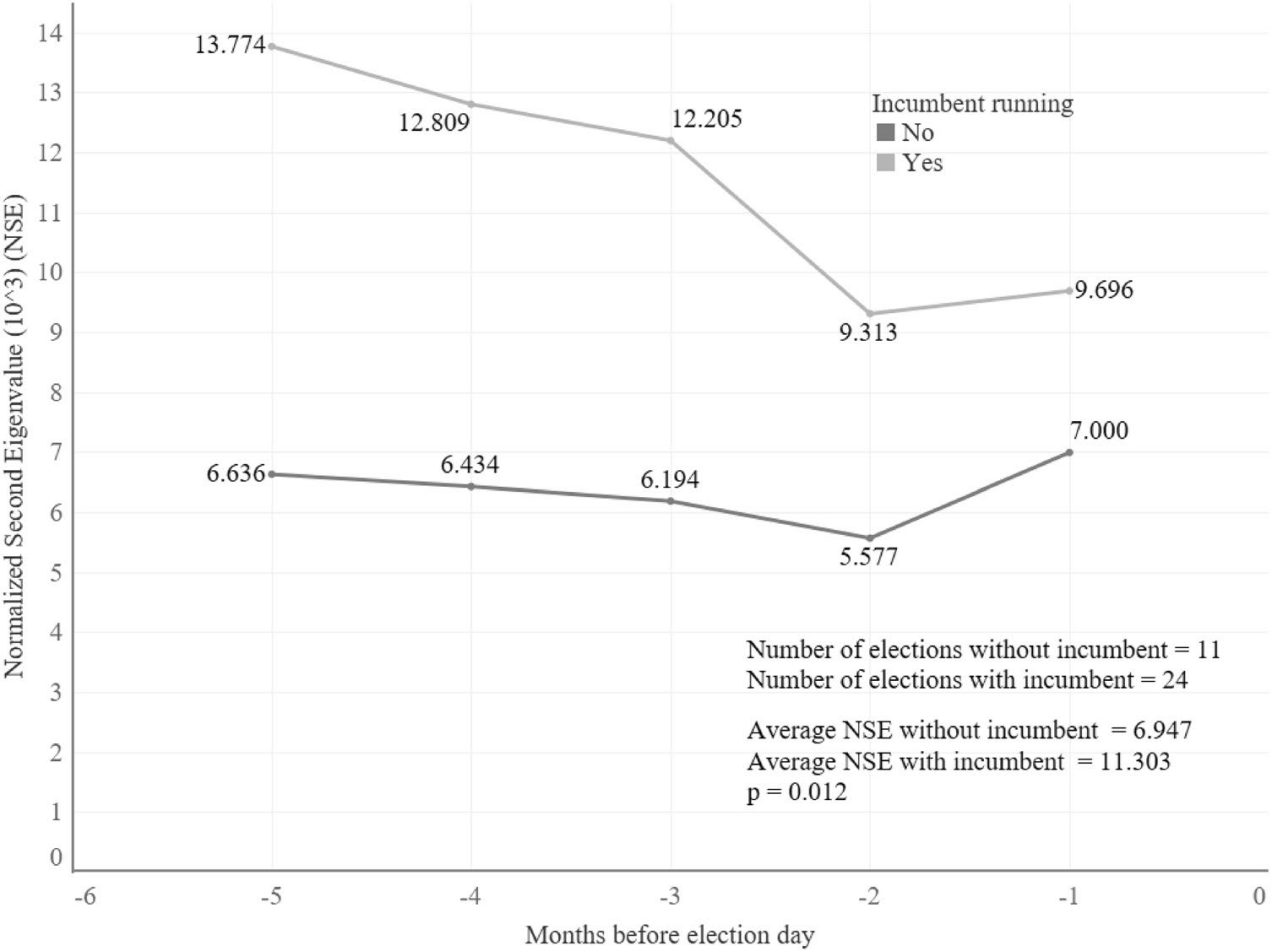
Evolution of the normalized second eigenvalue if the incumbent was or not among the election candidates. Since the normalized second eigenvalues do not have a normal distribution (its Kolmogorov-Smirnov test statistic (D) is 0.1951), a Mann–Whitney U Test was performed to determine if the differences between the means were statistically significant.

**Figure 4:**
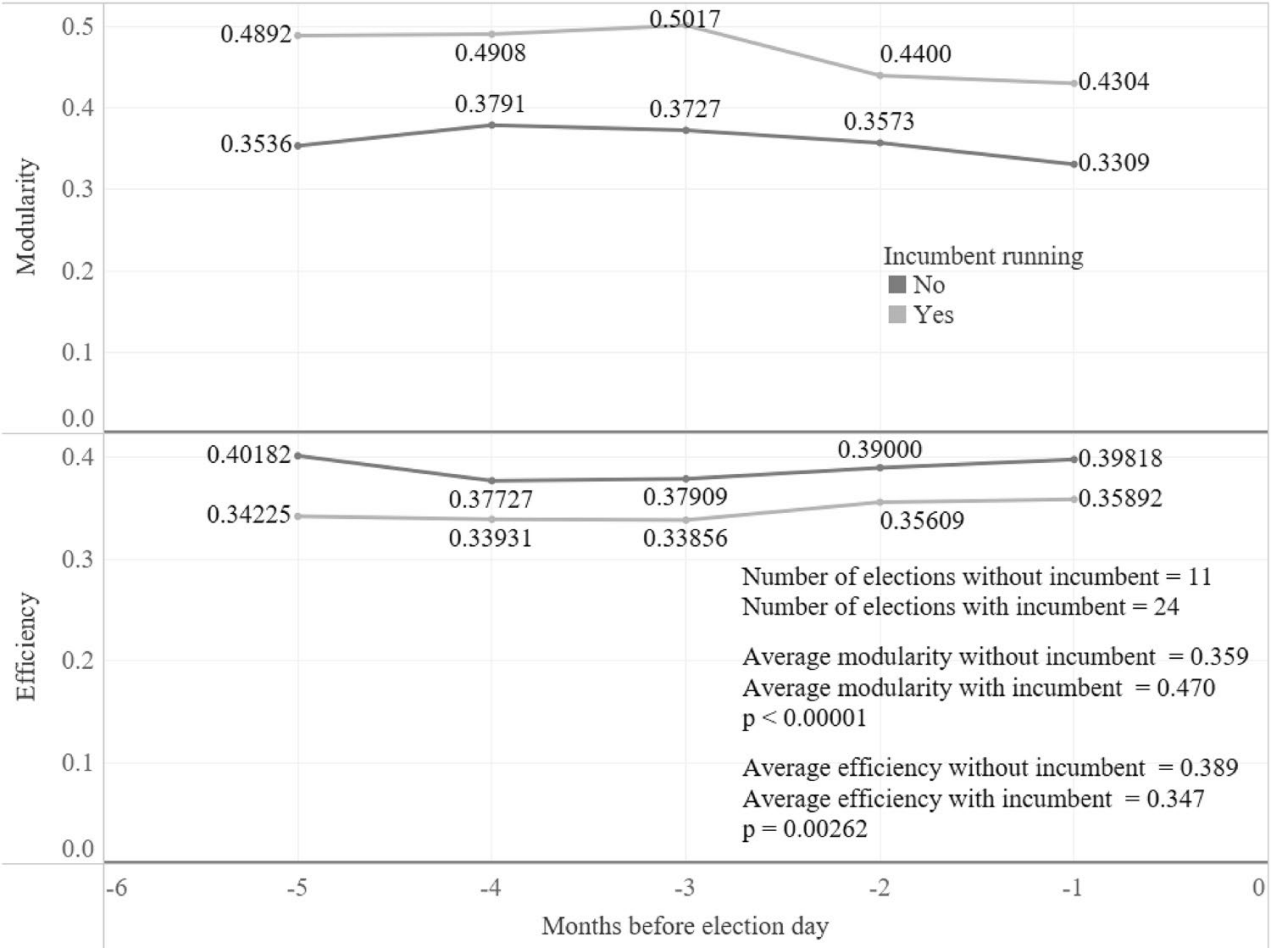
Evolution of modularity and efficiency according to whether the incumbent is running or not. A Mann–Whitney U Test was performed to determine if the differences between the means were statistically significant.

The elections were aggregated to test the two hypotheses previously established. For the first one, the results were contrary to the ones expected. In the five months before, the average second eigenvalue of the elections where the incumbent was running were larger, and statistically significant, than the ones where the incumbent was not on the ballot (Fig. 3). This discrepancy, however, does not prove the hypothesis wrong, rather it shows the way the conversations on this kind of election happens. As shown in Fig. 4, the efficiency of both groups is very similar, in contrast to the modularity where the elections with incumbents are always higher. This means that the difference relies on the fact that elections where the incumbent is running are more polarized and there are more closed groups tweeting about the same topic.

**Figure 5:**
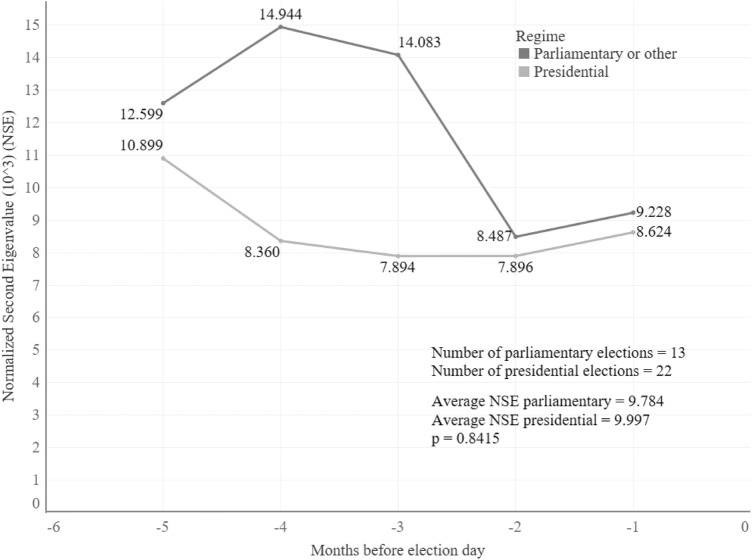
Evolution of the second eigenvalue according to the political regime. Since the normalized second eigenvalues do not have a normal distribution (its Kolmogorov–Smirnov test statistic (D) is 0.1951), a Mann–Whitney U Test was performed to determine if the differences between the means were statistically significant.

**Figure 6:**
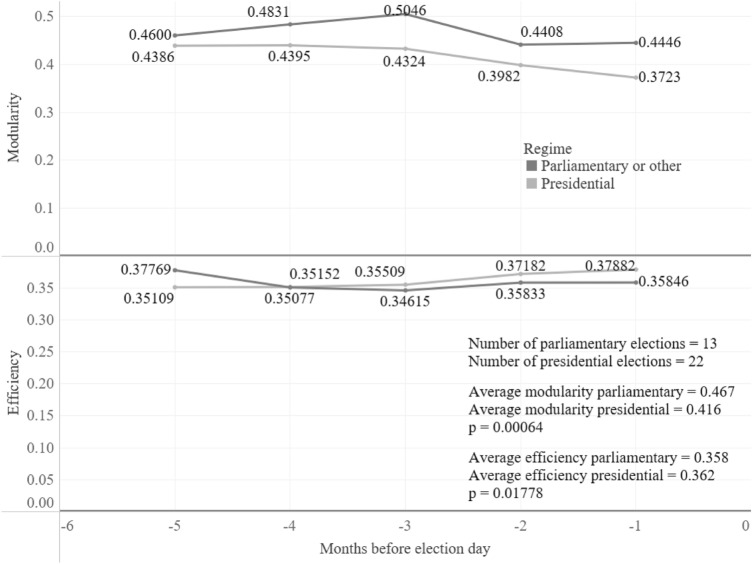
Evolution of modularity and efficiency according to the political regime. A Mann–Whitney U Test was performed to determine if the differences between the means were statistically significant.

In the same line, the second hypothesis did not seem to pan out as expected either. This hypothesis claimed that a parliamentary election had more topics on the public sphere (personality of the leader and party factors) than a presidential election (only personality of the leader), therefore, it was possible to have more conversations with other political factions. Figure 5 shows that parliamentary elections have a slower convergence than presidential elections, however, the differences are not statistically significant. Even though differences are not significant, which could be because in the last two months both regimes have a similar speed of convergence, there are dissimilarities on modularity and efficiency (see Fig. 6). Presidential elections tend to reduce their modularity and increase efficiency, especially in the two months prior to election day. In the end, this means that for parliamentary elections there are closer communities with fewer connections between them and the opposite is true for the presidential elections.

## Discussion and conclusion

Our research intended to bridge a gap between two areas of literature in Twitter studies: disinformation studies and electoral studies. The first area of literature focuses mostly on the effect of coordinated behavior on the aggregated patterns of the network, while the second one addresses how relevant the information from social networks can be used to better understand electoral processes. We have shown that an easily implementable framework, based on studies of convergence and spectral network theory, describes the process by which a society converges on an electoral outcome based on Twitter interactions. Furthermore, we have provided a comparative empirical analysis of the electoral processes in the democracies with the largest number of Twitter accounts. We have also addressed the most important variables addressed in mass media studies of electoral processes, mainly whether the incumbent is running and regime type^32^. Our results suggest that using this framework enables a novel understanding of electoral processes that can describe social phenomena in near real-time, well beyond inquiring whether social media are analogous to polling data.

Convergence depends on the structure and the connections of the network. As seen before, the second eigenvalue fluctuates according to these two measures. In election terms, to maintain a healthy democracy, where different points of view can be expressed and seen by others, an effective and fast diffusion of information is needed or, in other words, a faster convergence is desirable. Even though these two measures do not capture completely the variation of the second eigenvalue, it is useful to describe how conversations happen on Twitter and compare between different contexts.

The comparative analysis done with two of the classical characteristics of elections show different patterns on the conversation in the social network. This reflects the importance of institutional settings on the discussions that citizens have around the candidates and how they can influence the formation of communities or echo chambers before the election. The comprehension of these patterns is useful since it can help measure how healthy the political conversations on social media are (if different points of view are being contrasted). Evidence of this is that, even though the proposed hypothesis did not reflect the expected behavior, it was possible to see why this happened and make a clear profile of each group.

This paper builds on the literature on social media and elections as it creates a framework to analyze conversations to make them comparable between contexts and a useful indicator to understand its general behavior, thus going beyond the usual research done in electoral studies, which is usually focused on a single context, mostly descriptive or aimed at replacing polls.

Future studies on this subject should consider that computational hazards and Twitter biases affect the analyses that can be done. On the one hand, the criteria used to download the tweets meant that, in certain elections, millions of messages were downloaded for each month, leading to a massive network that the server could neither read nor calculate the second largest eigenvalue. To avoid this issue, smaller time spans can be used, although it is possible that each data point is more susceptible to contingent election events that can add noise to the comparison with other contexts. On the other hand, Twitter’s algorithm facilitates polarization by increasing the probability of showing a user messages that are in line with their views^30^. In terms of the framework, this means that all mentions are not shown to every user, which could lead to biases especially on larger networks. Furthermore, smaller networks could show a differentiated behavior from larger ones because of the amount of information that every individual can have about the conversation (in small settings all points of view could be seen). Avoiding this issue requires that elections with similar numbers of nodes and tweets are compared. This last point was not made in this study in order to have a bigger sample to test the hypothesis.

An interesting line of work derived from our findings, which was beyond the scope of our study, would be to explore the speed of convergence of a single election, and to identify perturbations of convergence in near real-time analysis. This could be one among other methods to identify massive disinformation campaigns during electoral processes, a matter of national security and a current threat to most democracies^1^^4^. Our study provides a baseline to identify convergence by regime type and involvement of the incumbent State leader.

## Data and methods

### Definition of key concepts

#### Modularity

Modularity is a quality index for clusterings^33^ natural divisions of network nodes into densely connected subgroups^34^, According to Network Science Barabasi’s Book, Networks with high modularity have dense connections between the nodes within modules but sparse connections between nodes in different modules.

#### Efficiency

The efficiency of a network is a measure of how efficiently it exchanges information, is a physically grounded and more general way to characterize networks displaying the small-world property^35,36^. It is inversely proportional to the distance, In this way, to greater distance between two nodes less efficiency, for instance if the distance between two nodes is 1 the efficiency is 1, while, if the distance between two nodes is 2, the efficiency will be 0.5 thus, less.

#### Second eigenvalue

According with Golub and Jackson^37^ a key insight is that the convergence time of an iterated stochastic matrix is related to its second largest eigenvalue in magnitude. In The book Social and economic networks (2008) by Matthew Jackson the author featured the relationship between the second eigenvalue magnitude and the speed of convergence of a consensus.

### Selection of elections

The objective for the selection of the elections was to have a varied group of countries, however, Twitter usage is not the same in all contexts and it changes over the years. Therefore, a dual criterion of inclusion was used: countries with the most users in the world and countries with elections held in 2020 or 2021. For the first, since Twitter does not have an official statistic of the number of accounts in each country, some organizations report every year the 20 countries with the estimated highest usage of the platform https://datareportal.com/essential-twitter-stats. The last election of each country was used, regardless if it happened before 2020. For the second, using Wikipedia, a list of elections that happened around the world between 2020 and 2021 was used. For each of the 78 elections gathered, the official accounts of the three candidates with the most votes were searched and the countries where at least one of the candidates did not use Twitter were dropped. The elections where the server was not capable of calculating the second eigenvalue due to the large number of tweets were also dropped. In the end, 35 elections were selected to download the tweets. In the repository there is a list of all the elections considered.

### Criteria to download the tweets

As mentioned before and to make it as comparable as possible, the criterias used for downloading a tweet was if it mentioned at least one of the candidates and if it was written in the time frame between election day (the first election day in some cases) and 150 days earlier (five months before the election). Using mentions is not restricted to the ‘@’ symbol on the text. Twitter API also includes responding to other user messages where at some point one or more of the candidates was mentioned. On the other hand, a 150-day frame was chosen because it could give a good approach to the evolution of the conversation. All the tweets were downloaded using Twitter API on a Python script with the credentials for academic use provided by the platform. The code and tweet ids are available on GitHub: https://github.com/dcardenas11831/Twitter-elections-consensus.
